# Efficacy of measuring the invasive diameter of lung adenocarcinoma using mediastinal window settings: A retrospective study

**DOI:** 10.1097/MD.0000000000020594

**Published:** 2020-06-26

**Authors:** Tsuyoshi Uchida, Hirochika Matsubara, Yuichiro Onuki, Hiroyasu Matsuoka, Tomofumi Ichihara, Hiroyuki Nakajima

**Affiliations:** Department of General Thoracic Surgery, Yamanashi University, Yamanashi, Japan.

**Keywords:** clinical staging, consolidation to maximum tumor diameter ratio, ground glass nodule, segmentectomy

## Abstract

The recently published 8th edition of the tumor node and metastasis Classification of Lung Cancer proposes using the maximum dimension of the solid component of a ground glass nodule (GGN) for the T categorization. However, few studies have investigated the collection of this information when using mediastinal window settings. In this study, we evaluated tumor measurement data obtained from computed tomography (CT) scans when using mediastinal window settings.

This study included 202 selected patients with persistent, partly solid GGNs detected on thin-slice CT after surgical treatment between 2004 and 2013. We compared the differences in tumor diameters measured by 2 different radiologists using a repeated-measures analysis of variance. We divided the patients into 2 groups based on the clinical T stage (T1a+T1b vs T1c) and estimated the probability of overall survival (OS) and disease-free survival (DFS) using Kaplan–Meier curves.

The study included 94 male and 108 female patients. The inter-reviewer differences between tumor diameters were significantly smaller when the consolidation to maximum tumor diameter ratio was ≤0.5. The 2 clinical groups classified by clinical T stage differed significantly with respect to DFS when using the mediastinal window settings. However, no significant differences in OS or DFS were observed when using the lung window setting.

Our study yielded 2 major findings. First, the diameters of GGNs could be measured more accurately using the mediastinal window setting. Second, measurements obtained using the mediastinal window setting more clearly depicted the effect of clinical T stage on DFS.

## 1 Introduction

An accurate evaluation of the clinical characteristics of lung cancer is an important step before determining the appropriate treatment approach. The 8th edition of the tumor node and metastasis (TNM) Classification of Lung Cancer was published on January 1, 2017. This revised classification proposes the use of the maximum dimension of the solid component of a ground glass nodule (GGN) to assign the T category. However, the maximum dimension of the ground glass component should also be recorded when assessing GGNs without a solid component or GGNs with a small consolidation to maximum tumor diameter ratio (CTR). Accordingly, the revised classification proposes the use of lung window settings rather than mediastinal window settings for clinical T staging.^[1]^ Ahn et al^[2]^ reported that the diameter of a GGN can be measured more accurately using mediastinal window settings. However, computed tomography (CT) measurements are related to pathological measurements determined using lung window settings,^[2]^ and there is no study on the relationship between prognosis and CT measurements in the literature. In this study, we investigated the potential interpretation of CT measurements obtained using mediastinal window settings as proposed by the revised TNM Classification of Lung Cancer.

## 2 Materials and methods

### 2.1 Patients

This was a retrospective study conducted at a single institution. Our institutional review board approved the study protocol and waived the requirement for informed patient consent.

### 2.2 Nodule selection and patients

We performed lobectomy in 408 cases of primary lung cancer between April 2004 and April 2013. The following inclusion criteria were used in this study: detection of a partly solid GGN on thin-slice CT sections (thickness, 1 or 1.25 mm) without enhancement; maximum tumor diameter ≤40 mm, which is classified as <cT2a in the 8th edition of the TNM Classification of Lung Cancer, and patients who underwent lobectomy and received a pathological diagnosis of adenocarcinoma. Partly solid GGNs were divided into 2 sections: a pure ground glass component with a CT value between –700 and –300 Hounsfield units (HUs) and a solid part with a CT value exceeding –300 HU. Based on these criteria, we selected 202 partly solid GGNs.

### 2.3 CT

CT scans were obtained using 1 of 2 CT scanners (Aquilion, TOSHIBA, Tokyo, Japan) available at the hospital. The following CT parameters were applied: section thickness, 1 or 1.25 mm; lung window width and level, 1500 and –600 HUs, respectively; and mediastinal window width and level, 320 and 60 HUs, respectively.

### 2.4 Analysis of CT images

We consulted 2 board-certified radiologists to analyze the CT images. One radiologist (A) had 5 years of independent experience in chest CT scan interpretation, and the other radiologist (B) had 20 years of experience in chest CT chest scan interpretation. Both radiologists reviewed thin-slice CT scans in the absence of patient data and independently measured the diameters of the solid parts (under the lung window settings) and the maximum diameters (under the mediastinal window settings).

### 2.5 Statistical analysis

**Figure 1:**
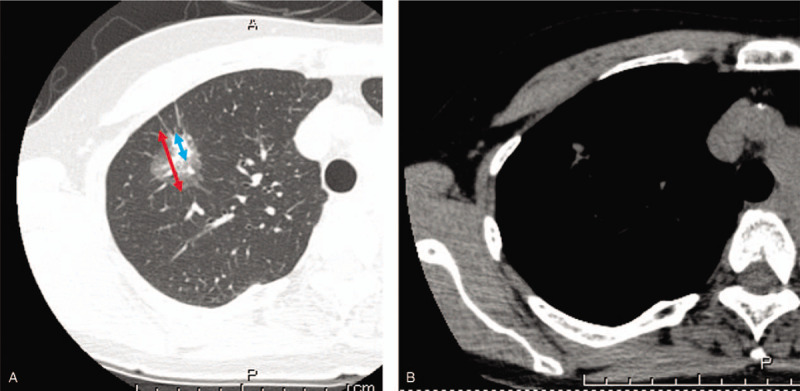
An example of a ground glass nodule measured using (A) the lung window setting or (B) the mediastinal window setting. The blue arrow indicates the diameter of the solid part of the tumor, while the red arrow indicates the maximum tumor diameter. Consolidation to maximum tumor diameter ratio (CTR) = blue arrow/red arrow.

The patients’ characteristics are reported as means with standard deviations for continuous variables and as numbers and frequencies for categorical variables. We used a repeated-measures analysis of variance to compare differences in the diameters measured by the 2 radiologists. The first analysis compared all cases, while the second analysis compared cases after classification according to the CTR,^[3]^ which was calculated as (diameter of the solid part)/(maximum tumor diameter). Figure 1 depicts a representative GGN and the protocol for determining CTR.

Overall survival (OS) was defined as the length of time from cancer diagnosis until the analysis of a living patient's data. Disease-free survival (DFS) was defined as the length of time from cancer diagnosis until cancer recurrence. We divided the patients into 2 groups based on the 8th edition of the TNM Classification of Lung Cancer, as follows: cT1a+T1b and cT1c (Table 1). Further, we used the Kaplan–Meier method to estimate the probabilities of OS and DFS and evaluated differences in survival between the cT stage groups using the log-rank test. All *P*-values were 2 sided, and a value <0.05 was considered significant. Statistical analyses were performed using the EZR 3.5.1 software program (Jichi Medical University Saitama Medical Center, Saitama, Japan).^[4]^

**Table 1:**
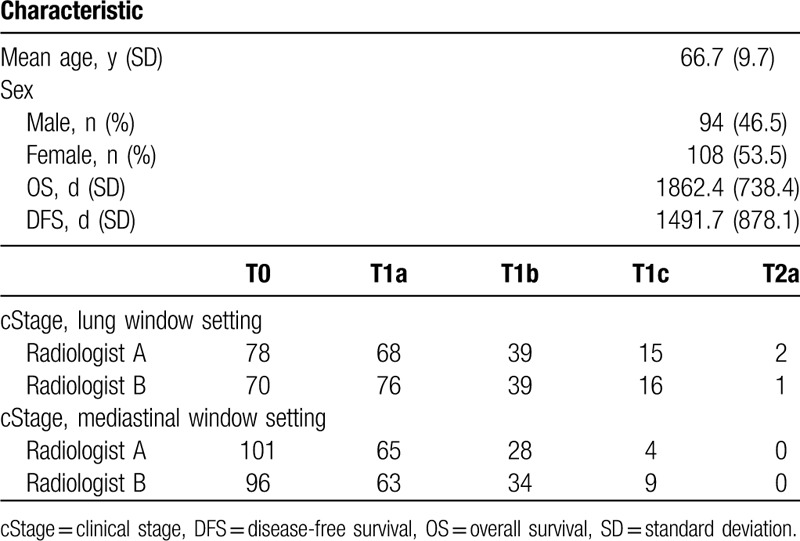
Patient background information and radiology measurements using the lung window settings or mediastinal window settings.

## 3 Results

Table 1 summarizes the patients’ background information and the results of the clinical stage measurements of nodules that were classified by the 2 radiologists (A and B) using different window settings. The patients had a mean age of 66 years, and 46.5% were men.

**Figure 2:**
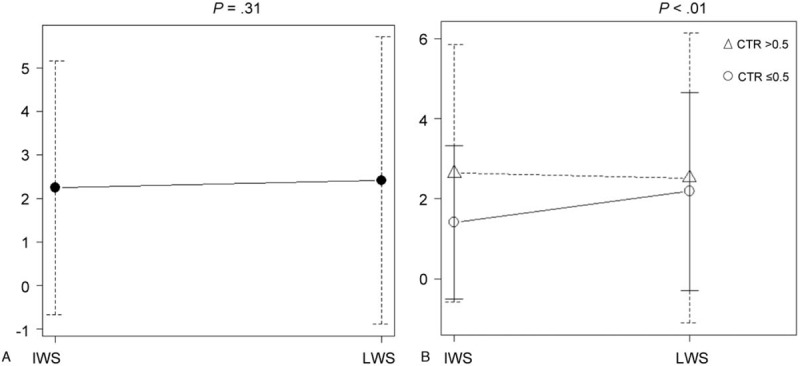
Differences in tumor diameters measured by 2 different radiologists. (A) In cases without classification by consolidation to maximum tumor diameter ratio (CTR), there were no significant differences between the measurements made by the radiologists (A and B). (B) The difference between the tumor diameters measured by the 2 radiologists was smaller in the group of lower CTRs (CTR ≤ 0.5) when measured using the mediastinal window setting. The differences were analyzed using ANOVA. ANOVA = analysis of variance.

Figure 2 presents the differences between the diameters measured by the 2 radiologists (i.e., observer differences). Before classification according to the CTR, no significant differences were observed between the diameters measured by radiologists A and B (Fig. 2A). However, after classification by the CTR, the differences in diameters between the 2 radiologists were smaller for nodules with lower CTR values (i.e., <0.5) when the measurements were determined using the mediastinal window setting (Fig. 2B).

**Figure 3:**
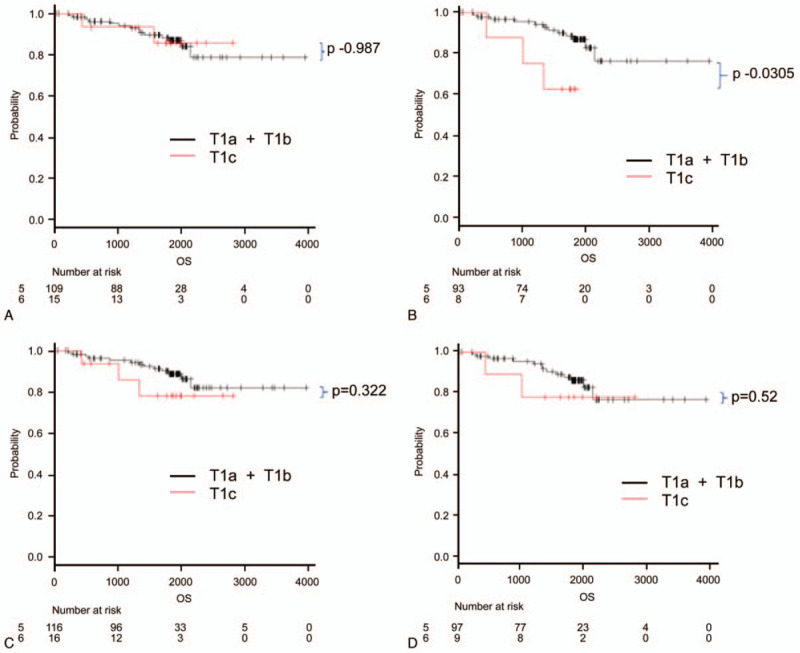
Overall survival divided by clinical T stage (T1a+T1b vs T1c). Graphs are based on measurements made by radiologist A using (A) lung window settings and (B) mediastinal window settings and on measurements made by radiologist B using (C) lung window settings, and (D) mediastinal window settings. The differences in survival were determined using a log-rank test, and the *P*-values are 2 sided.

**Figure 4:**
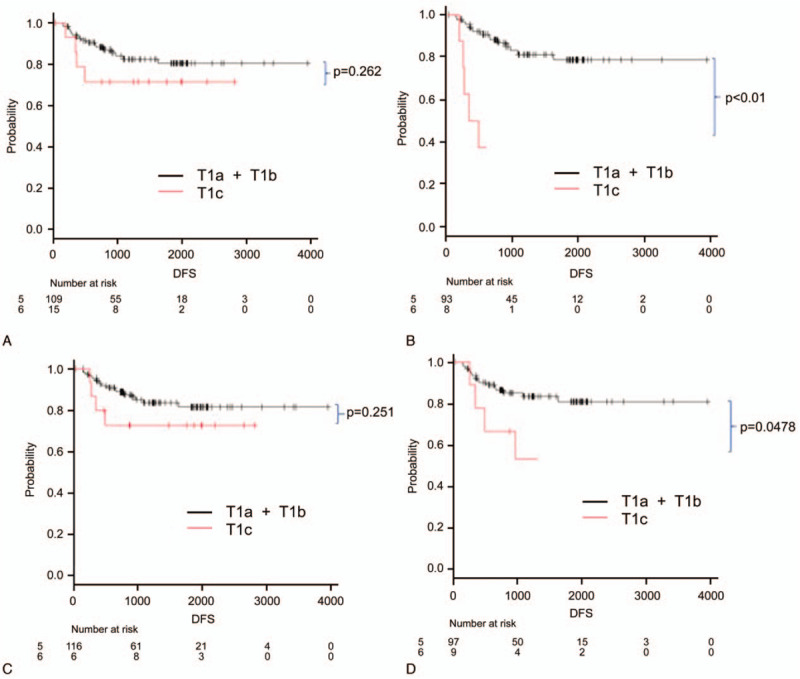
Disease-free survival divided by clinical T stage (T1a+T1b vs T1c). Graphs are based on measurements made by radiologist A using (A) lung window settings and (B) mediastinal window settings and on measurements made by radiologist B using (C) lung window settings and (D) mediastinal window settings. The differences in survival were determined using a log-rank test, and the *P*-values are 2 sided.

Figure 3 presents the comparison of OS according to the clinical T stage (T1a+T1b vs T1c). Figure 3A depicts the OS analysis of cases classified by the CTR as measured by radiologist A using the lung window setting, and Fig. 3B depicts a similar analysis by radiologist A using the mediastinal window setting. Figure 3C and D depicts the analysis described in Fig. 3A and B, respectively, using measurements determined by radiologist B. These comparisons did not reveal any significant differences. Figure 4A–D reveals similar analyses of DFS based on the same denominators. Although significant differences were observed between the 2 curves when using the mediastinal window setting, these differences were not significant when using the lung window settings.

## 4 Discussion

This study yielded 2 important findings, namely: the GGN diameter could be measured more accurately using the mediastinal window setting and the Kaplan–Meier curve of estimated DFS clearly demonstrated the difference in survival based on the clinical T stage. It is very important to accurately diagnose the degree of progression when treating tumors. Therefore, clinicians who measure tumors must attempt to reduce errors. However, the shadow of GGN spreads faintly, and its boundaries may be interpreted variably by different observers. Although previous studies reported that the use of semi-automatic measurements could guarantee accuracy,^[5]^ we hypothesized that even these semi-automatic conditions would vary, leading to variations in accuracy.

Some studies reported that CTR ≤ 0.5 is an important cutoff value for predicting pathologic invasiveness in lung adenocarcinoma.^[6–9]^ Therefore, we used CTR ≤ 0.5 as a cutoff value in this study. Notably, we observed smaller differences in observer measurements within the group of nodules with smaller CTRs, and this result was dependent on the interpretation of the GGN component. We further determined that the mediastinal window setting enabled us to measure the tumor more accurately when the GGN was large.

Although lung cancer treatment is administered with the goal of extending OS, our analysis revealed no significant differences in OS between the clinical T stage groups according to the radiologist or window settings. DFS is an indicator of sufficient resection, and in our study, the mediastinal window setting yielded more clearly drawn DFS curves and significant differences between the 2 study groups, irrespective of the radiologist who determined the measurements. This finding suggests that we were better able to identify patients with clinical stage I disease who had a good survival prognosis. The Japan Clinical Oncology Group (JCOG) 0201 study reported that patients with an excellent prognosis after diagnosis presented with lung adenocarcinoma lesions with whole diameter ≤3.0 cm and CTR ≤ 0.5.^[10,11]^ Our results confirmed those earlier findings and further demonstrated a low recurrence rate in cases with tumors measuring ≤2.0 cm using the mediastinal window setting.

This study had some limitations. First, the retrospective study design led to the possibility of a selection bias. Second, we did not investigate pathological factors that could influence the T stage such as a pleural invasion. Third, we included a relatively small number of cases obtained at a single institution.

In conclusion, although the JCOG 0802 study suggested that segmentectomy could be considered a standard treatment for lung cancer,^[12]^ the risk of local recurrence after treatment with a limited resection method, such as segmentectomy, should be noted. Our study findings demonstrate that tumors with a measurement of 2.0 cm based on the mediastinal window setting are associated with a low rate of recurrence. This observation may influence the indication of segmentectomy for clinical stage I lung adenocarcinoma.

## Acknowledgments

The authors would like to thank Editage (www.editage.com) for English language editing. They also wish to thank Eiichi Sawada and Tatsuya Shimizu, the radiologists who participated in this study, for their valuable contributions.

## Author contributions

**Conceptualization:** Tsuyoshi Uchida, Hirochika Matsubara.

**Data curation:** Tsuyoshi Uchida, Hiryoasu Matsuoka.

**Formal analysis:** Tsuyoshi Uchida, Yuichiro Onuki, Hiroyasu Matsuoka.

**Investigation:** Tsuyoshi Uchida.

**Methodology:** Hiroyasu Matsuoka, Hirochika Matsubara.

**Project administration:** Tsuyoshi Uchida, Hirochika Matsubara, Yuichiro Onuki, Hiroyasu Matsuoka, Tomofumi Ichihara, Hiroyuki Nakajima.

**Resources:** Tsuyoshi Uchida.

**Software:** Hiroyasu Matsuoka.

**Validation:** Tsuyoshi Uchida, Hirochika Matsubara, Yuichiro Onuki, Hiroyasu Matsuoka, Tomofumi Ichihara, Hiroyuki Nakajima.

**Writing – original draft:** Tsuyoshi Uchida.

**Writing – review & editing:** Tsuyoshi Uchida, Hirochika Matsubara, Yuichiro Onuki, Hiroyasu Matsuoka, Tomofumi Ichihara, Hiroyuki Nakajima.
